# Cysteine-rich intestinal protein 1 suppresses apoptosis and chemosensitivity to 5-fluorouracil in colorectal cancer through ubiquitin-mediated Fas degradation

**DOI:** 10.1186/s13046-019-1117-z

**Published:** 2019-03-08

**Authors:** Lanzhi Zhang, Rui Zhou, Weibin Zhang, Xueqing Yao, Weidong Li, Lijun Xu, Xuegang Sun, Liang Zhao

**Affiliations:** 1 Department of Pathology, Nanfang Hospital, Southern Medical University, Guangzhou, 510515 Guangdong China; 2 Department of Pathology, School of Basic Medical Sciences, Southern Medical University, Guangzhou, Guangdong China; 3 Guangdong Provincial Key Laboratory of Molecular Oncologic Pathology, Southern Medical University, Guangzhou, China; 4 Department of General Surgery, Guangdong General Hospital, Guangdong Academy of Medical Science, Guangzhou, Guangdong China; 5 Department of Medical Oncology, Affiliated Tumor Hospital of Guangzhou Medical University, Guangzhou, China; 6 School of Traditional Chinese Medicine, Southern Medical University, Guangzhou, 510515 China

**Keywords:** Cysteine-rich intestinal protein 1, Colorectal cancer, Apoptosis, Chemoresistant, FAS

## Abstract

**Background:**

Cysteine-rich intestinal protein 1 (CRIP1) is highly expressed in human intestine and aberrantly expressed in several types of tumor. However, studies on CRIP1 are limited and its role on tumor development and progression remains controversial and elusive.

**Methods:**

Immunohistochemistry was performed to evaluate the expression of CRIP1 in paired normal and colorectal tumor specimens, as well as colorectal cell lines. Functional assays, such as CCK8, TUNEL assay and in vivo tumor growth assay, were used to detect the proliferation, apoptosis and response to 5-FU of CRIP1. Western blot was used to analyze Fas-mediated pathway induced by CRIP1. Rescue experiments were performed to evaluate the essential role of CRIP1 for Fas-mediated apoptosis.

**Results:**

We demonstrated that CRIP1 is overexpressed in CRC tissues compared with adjacent normal mucosa. CRIP1 could dramatically recover the 5-Fluorouracil (5-FU) inhibited CRC cell proliferation in vitro and stimulate the tumor formation of CRC in vivo, probably through inhibiting CRC cell apoptosis. Moreover, CRIP1 also dramatically recovered the 5-Fluorouracil (5-FU) induced tumor cell apoptosis in vitro*.* Further study demonstrated that CRIP1 down-regulated the expression of Fas protein and proteins related to Fas-mediated apoptosis. CRIP1 could interact with Fas protein and stimulate its ubiquitination and degradation. In addition, a negative correlation was detected between the expression of CRIP1 and Fas protein in most of the clinical human CRC samples.

**Conclusion:**

The current research reveals a vital role of CRIP1 in CRC progression, which provide a novel target for clinical drug resistance of colorectal cancer and undoubtedly contributing to the therapeutic strategies in CRC.

**Electronic supplementary material:**

The online version of this article (10.1186/s13046-019-1117-z) contains supplementary material, which is available to authorized users.

## Introduction

Colorectal cancer (CRC) ranks third in terms of incidence but second in terms of mortality [1]. Although numerous efforts have been made to improve diagnostic and therapeutic strategies for CRC patients, survival rate of patients with advanced colorectal cancer remains low within five years [2]. Most patients still die due to the therapeutic resistance of CRC to conventional anti-cancer drugs and recurrence after resection. The internationally accepted first-line treatment for metastatic colorectal cancer (mCRC) is FOLFOX or FOLFIRI treatment regimen consists of 5-fluorouracil (5-FU)/leucovorin (LV) plus oxaliplatin or irinotecan [3]. 5-FU, the cornerstone of CRC chemotherapy, could stop the DNA production of tumor cells through blocking the action of thymidylate synthase. Therefore, it is urgent to uncover vital molecular mechanisms underlying CRC progression and drug resistance, which helps to figure out novel diagnostic and prognostic biomarkers.

Cysteine-rich intestinal protein1 (CRIP1) is a member of LIM/double-zinc finger protein family predominantly expressed in the intestine, which is first verified important for zinc transport and absorption [4]. Besides intestine, CRIP1 is subsequently recognized in other organs including colon, lung, spleen, thymus and head in transgenic mice [5]. CRIP1 was further detected in immune cells in tissues of rats, suggested the involvement of this protein in host defense [6]. Aberrant expression of CRIP1 was also mentioned in several tumor types including prostate cancer, pancreatic caner, cervical cancer, breast cancer, osteosarcoma, gastric cancer, and thyroid cancer [7–13]. However, related studies are very limited and the role of CRIP1 is controversial in different tumor types. High expression of CRIP1 is correlated with a favorable prognosis in osteosarcoma and breast cancer [10, 11]. In contrast, high CRIP1 expression was confirmed as a novel and independent prognostic factor for poor prognosis in gastric cancer patients [12]. Knockdown of CRIP1 inhibited the proliferation of thyroid carcinoma cells through inducing G1 arrest and apoptosis, while silencing of CRIP1 significantly elevated the proliferation of T47D and BT474 breast cancer cells via reducing the phosphorylation of cdc2. In addition, knockdown of CRIP1 increased breast cancer cell invasion in vitro [10]. CRIP1 was also identified as a bone specific breast cancer metastasis gene [14, 15]. Except thoes functional stdudies mentioned above, mechanisms under CRIP1 mediated tumor devlopment and progression are largely unknown.

As few data is available on CRIP1 in colorectal cancer, this study was undertaken to systematically characterize the expression and functions of CRIP1 during CRC development and progression. Our results suggest that CRIP1 contribute to the proliferation and chemosensitivity of colorectal cancer cells through inhibiting Fas signaling cascade related apoptosis. Furthermore, the relationship between CRIP1 and Fas expression was explored for the first time in the clinical tissues of CRC patients. The presented findings revealed a novel role of CRIP1 on the progression and chemosensitivity of colorectal cancer.

## Materials and methods

### Cell lines and cell culture

The normal human colon epithelial cell line NCM460 and CRC cell lines including LS174T, RKO, HT29, HCT116, SW480 and SW620 were obtained from the Cell Bank of the Chinese Academy of Sciences (Shanghai, China) and maintained as previously described [16]. All the cells were cultured in RPMI 1640 (Hyclone, Logan, Utah, USA) supplemented with 10% fetal bovine serum (FBS) (Gibco-BRL, Invitrogen, Paisley, UK) at 37 °C with a humidity of 5% CO_2_. MG132 (Cell Signaling Technology, DM, USA), which reduces the degradation of ubiquitin-conjugated proteins, was first diluted in DMSO and further diluted in medium containing bovine serum albumin and added to CRC cells at a final concentration of 30 μM for 24 h. Plasmid pcDNA3-CRIP1 was constructed in our lab and CRIP1 specific siCRIP1 was purchased from GenePharma (Shanghai, China). CRC cells at exponential growth phase were plated into 6-well plates for 24 h at a density of 0.5 × 10^5^ cells/mL, and transfected with 1 mg of siRNA or 4 μg cDNA in reduced serum medium (OPTI-MEM-I; Invitrogen) using Lipofectamine 3000 transfection reagent (Life Technologies, Carlsbad, USA), according to the manufacturer’s protocol.

### Tissue samples

Fresh and paraffin-embedded tissue specimens were provided by the Tumor Tissue Bank of Nanfang Hospital. In each case, a diagnosis of primary CRC had been made. The study was approved by the Ethics Committee of Southern Medical University and all aspects of the study comply with the Declaration of Helsinki. Ethics Committee of the Southern Medical University specifically approved that not informed consent was required because data were going to be analyzed anonymously.

### Animals

All animal experiments were carried out with the approval of the Southern Medical University Animal Care and Use Committee in accordance with the guidelines for the ethical treatment of animals. Nude nu/nu mice were maintained in a barrier facility in racks filtered with high-efficiency particulate air filter. All animal experiments involved ethical and humane treatment under a license from the Guangdong Provincial Bureau of Science [17].

### Western blot analysis

Immunoblot analysis was performed to assess protein expression of cell lysates (20-60 μg) in RIPA buffer as previously described [16]. Antibodies of Apoptosis Antibody Sampler Kit (Cat No. 9930), FasL (Cat No.4237) and β-tubulin (Cat No. 2146) were purchased from Cell Signaling Technology (Danvers, MA, USA); Antibodies to CRIP1 (Cat No. ab185558), Fas (Cat No. ab133619) and Ubiquitin (Cat No. ab140601) were purchased from Abcam (Cambridge, MA, USA); Fluorescent secondary antibodies and HRP-conjugated antibodies were obtained from Zhongshanjinqiao Biotechnology (Beijing, China).

### RNA extraction and real-time PCR

Real-time PCR was performed in a LightCycler 480 thermal cycler (Roche) as described previously [18]. Total RNAs were isolated from CRC cell lines with Trizol reagent (Invitrogen Life Technologies, Gaithersburg, MD) according to manufacturer’s protocol. Reagents were purchased from TAKARA (5 × PrimeScript RT Master Mix, RR036A, SYBR® Premix Ex Taq™ II, RR820A). GAPDH was chosen as an internal quantitative control. All the primers were purchased from Invitrogen and the primer sequences were shown as Additional file 1: Table S1. The expression level of each targeted gene was normalized as fold change compared with control or reference group. Fold changes were calculated through relative quantification (2^-∆∆*CT*^) [19].

### Cell viability assay

Cell viability was quantified using the Cell Counting Kit-8 (CCK-8; Dojindo, Kumamoto, Japan) as described previously [18]. Cell proliferation was assessed using 5-ethynyl-20-deoxyuridine (EdU) incorporation assay (Kaijishengwu, China, KGA331–100) according to the manufacturer’s instructions. In brief, cells were seeded into 6-well plates at a concentration of 5 × 10^3^ cells per well for 24–48 h and then exposed to EdU (50 μM) for 1–1.5 h at 37 °C. Subsequently, the cells were fixed with 4% paraformaldehyde and then permeabilized using 0.5% Triton X-100. Finally, the cells reacted with 100 μl of 1 × Apollo® reaction cocktail for 30 min, followed by incubation with 100 μl of DAPI (5 μg/mL). The percentage of EdU-positive cells was calculated as [(EdU add-in cells/Hoechst-stained cells) × 100%] in five arbitrarily selected fields from each group [20].

### Apoptosis assay

Propidium iodide (PI) and Annexin V-FITC-flow cytometry assay (BD Pharmingen, CA) was used to detect the apoptosis in the cells. Cells were treated with 5-fluorouracil (5-FU) for 18–24 h and harvested in PBS. Then the cells were re-suspended in binding buffer (BD Pharmingen, CA), and stained with FITC-conjugated annexin V and PI (Kaijishengwu, China, KGA107). After staining, the cells were analysed by flow cytometry (FACS Calibar; Becton-Dickinson) using Cell Quest software [21].

### TUNEL assay

TUNEL assay was processed using the in situ cell death detection kit (Roche; Mannheim, Germany). After washing with PBS for three times, treated cells were permeabilization with proteinase K for 10 min. The cells were then incubated in TdT buffer at 37 °C for 1 h after another three times of washing. Afterward, the cells were incubated with primary antibody CRIP1 overnight at 4 °C, followed by the incubation of PE-conjugated secondary antibodies and DAPI counterstaining. The apoptosis index was calculated by counting the number of TUNEL/CRIP1-positive cells using a fluorescence microscope (Olympus IX73, Japan).

### In vivo tumour growth assay

Approximately 2 × 10^6^ SW620-Vector, SW620-CRIP1, HCT116-shNC and HCT116-shCRIP1 cells were injected subcutaneously in left and right flank of 4- to 6-week-old Balb/C- nu/nu athymic nude mice (*n* = 6 per group) obtained from Animal Center of Southern Medical University. Tumor size was measured by a slide caliper, and tumor volume was also calculated. The experiments were performed according to institutional guidelines and approved by the Institution Animal Care and Use Committee of Southern Medical University.

### Immunoprecipitation

The cells were harvested and washed with chilled PBS for three times, then lysed in IP Lysis Buffer containing 2 mM Tris–HCl (pH 7.4), 10 mM EDTA, 100 mM NaCl and 1% Octylphenoxypolyethoxyethanol (IGEPAL). The protein was quantified using a BCA Protein Assay Kit (KeyGen, Biotechnology, China), then diluted into 1 mg/ml with chilled wash buffer (with protease and phosphatase inhibitor). Cell lysates were subjected to immunoprecipitation with antibodies against CRIP1 or Fas on protein A/G beads (Santa Cruz Biotechnology). Protein mixture was heat denatured in 5% SDS-PAGE sample loading buffer, and then subjected to western blot assay.

### Statistical analysis

Data were analyzed using SPSS version 19.0 software (SPSS; Chicago, USA). The Student *t*-test and the one-way ANOVA test were carried out for qRT-PCR, EdU, apoptosis rate and TUNEL analysis. CCK-8 analysis was applied to calculate the tumor growth curve. Significance of correlation between the expression of CRIP1/Fas and histopathological factors were determined using Pearson’s chi-squared (χ 2) test. Statistical significance was established at *P* < 0.05.

## Results

### CRIP1 is overexpressed in CRC tissues and cell lines

**Fig. 1:**
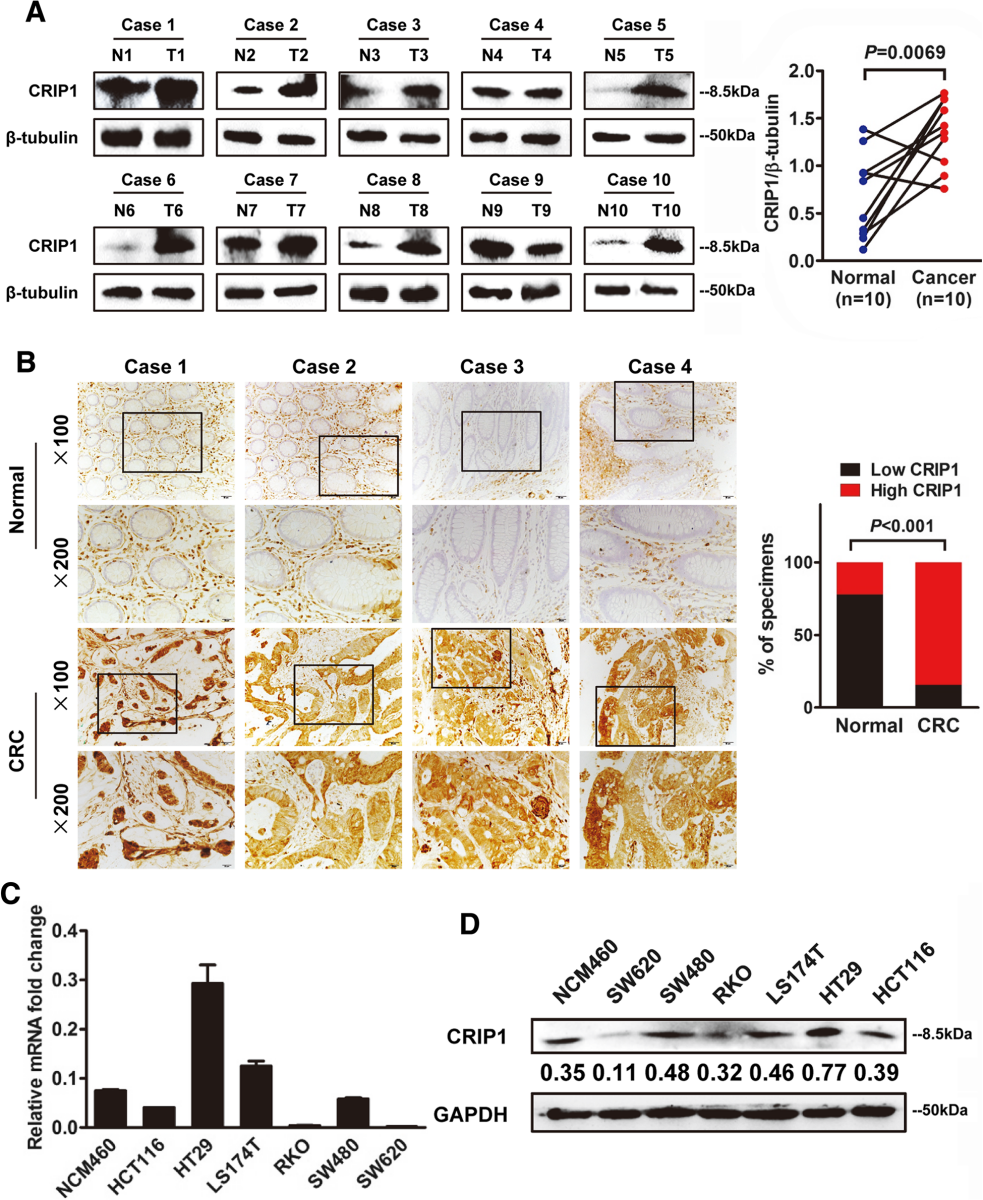
CRIP1 is overexpressed in both CRC tissues and cell lines. **a**. Left panel: Western blot assay for CRIP1 expression in ten CRC tissues (T) and matched normal adjacent normal tissues (N) from the same patient. Right panel: Quantification of CRIP1 expression in CRC tissues and adjacent normal tissues of ten patients. **b**. Left panel: IHC staining indicated that CRIP1 protein expression was increased in human CRC compared with normal intestinal epithelium (four representative slides was shown). Right panel: Graphical illustration of statistical CRIP1 distribution in CRC tissues. CRIP1 is significantly highly expressed in CRC tissues compared with that in adjacent non-tumorous tissue. **c**. Real-time PCR analyzed the expression of CRIP1 in NCM460 and six different CRC cell lines. The values represented relative CRIP1 level after normalization to the expression of β-tubulin (means ±SD, *N* = 3) **d**. Western blot analysis for the expression of CRIP1 in NCM460 and CRC cell lines. Immunosignals were quantified by densitometric scanning

Western blot assay was used to detect the expression of CRIP1 in both CRC tissues and cell lines. We measured the expression of CRIP1 in ten pairs of fresh primary CRC tissues with adjacent normal colorectal mucosa. As shown in Fig. 1a, a higher CRIP1 expression was observed in 8 of 10 CRC cases compared with matched adjacent normal tissues (*P* = 0.0069). Furthermore, we measured the expression of CRIP1 in paraffin-embedded CRC tissues by immunohistochemistry (IHC). Our data indicated that CRIP1 was overexpressed in both the cytoplasm and the nucleus of CRC cells, and its expression could be detected in the mesenchyme as well (Fig. 1b, left panel). Moreover, CRIP1 was significantly up-regulated in 73.68% (28/38) of CRC tissues examined compared to the adjacent normal tissues (*P* < 0.001, Fig. 1b, right panel). Real-time PCR and western blot were performed to analyze the expression of CRIP1 in different CRC cell lines. Because the origin and biological background of each cell line is distinctive, the expression pattern of CRIP1 mRNA and protein is also different among all cell lines. CRIP1 is obviously overexpressed in HT29 and LST174 CRC cells compared with that in normal mucosa epithelium cell line NCM460 at the transcriptional level, whereas it has a relatively lower expression in RKO and SW620 cells (Fig. 1c). Consistently, similar results were obtained in the following western blot assay (Fig. 1d). CRIP1 highly expressed SW480 and HCT116 cells were selected to perform loss-of-function assay, whereas CRIP1 lowly expressed RKO and SW620 cells were chosen to carry out gain-of-function assay. HT29 and LST174 cells were not included for their low transfection efficiency and inert biological behavior.

### Overexpression of CRIP1 recovers 5-FU suppressed SW620 cell proliferation both in vitro and in vivo

**Fig. 2:**
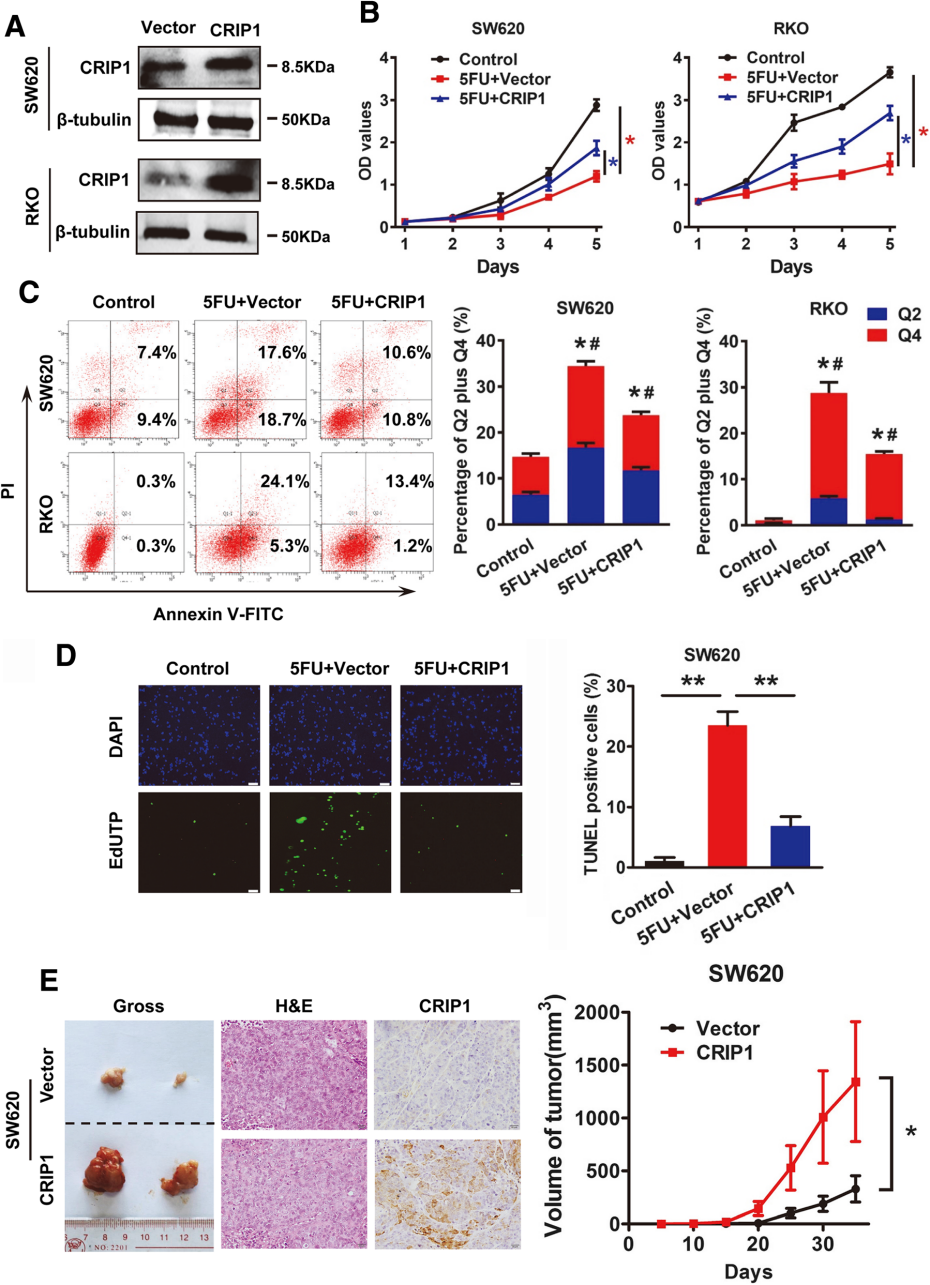
Overexpression of CRIP1 recovers 5-FU suppressed CRC cell proliferation both in vitro and in vivo. **a**. Western blot assay was used to detect the overexpression of CRIP1 in SW620 and RKO cells. **b**. CCK8 assay was used to study the effects of CRIP1 on 5-FU inhibited CRC cell proliferation. **c**. Annexin V-FITC/PI flow cytometry assay showed the effects of CRIP1 on 5-FU induced apoptosis. Bars on the right panel represent percentage of cells in Q2 + Q4. **d**. TUNEL assay showed the effects of CRIP1 on 5-FU induced apoptosis of CRC cells. Bars on the right panel represent the percentage of TUNEL positive cells. **e**. IHC staining showed the expression of CRIP1 in subcutaneous tumors formed by CRIP1 overexpressed CRC cells. Right panel represents the growth pattern of subcutaneous tumors

To evaluate the effects of CRIP1 on CRC cell proliferation, we transfected SW620 and RKO cells with CRIP1 overexpression vector. The transfection efficiency was monitored by western blot assay (Fig. 2a). We performed cell counting kit (CCK8) assay to evaluate the role of CRIP1 on CRC cell proliferation in vitro. As shown in Fig. 2b, CRIP1 dramatically restored the proliferation of SW620 and RKO cells inhibited by the treatment of 5-Fluorouracil (5-FU) (*P* < 0.05). Similarly, 5-ethynyl-2′-deoxyuridine (EdU) incorporation assay showed the effect of CRIP1 on accelerating DNA synthesis. However, no obvious changes in cell cycles were observed in CRIP1 overexpressed SW620 cells (Additional file 1: Figure S1). In order to verify whether the CRIP1 stimulated cell proliferation is performed through reducing cell apoptosis, we performed Annexin V-FITC/PI flow cytometry assay and TUNEL staining assay to detect cell apoptosis. 5-Fluorouracil (5-FU) induced the basal cell apoptosis level of SW620 and RKO cells. Flowcytometry data indicated that CRIP1 significantly reduced the apoptosis rate induced by 5-FU (Fig. 2c). Consistently, apoptosis cells labeled by TUNEL assay in green fluorescence dramatically decreased in CRIP1 overexpressed SW620 cells group (*P* < 0.05; Fig. 2d).

To investigate the biological effect of CRIP1 on CRC cells proliferation in vivo, we used a lentivirus-based system to establish CRIP1 stable overexpressing CRC cells. Results indicated that CRIP1 could significantly stimulate the growth of subcutaneous tumors and the IHC signal of CRIP1 is dramtically strong in the subcutaneous tumors formed by the CRIP overexpressing group compared with that formed by the control group (Fig. 2e, Additional file 1: Figure S2, left panel).

### Knockdown of CRIP1 aggravates 5-FU suppressed HCT116 cell proliferation both in vitro and in vivo

**Fig. 3:**
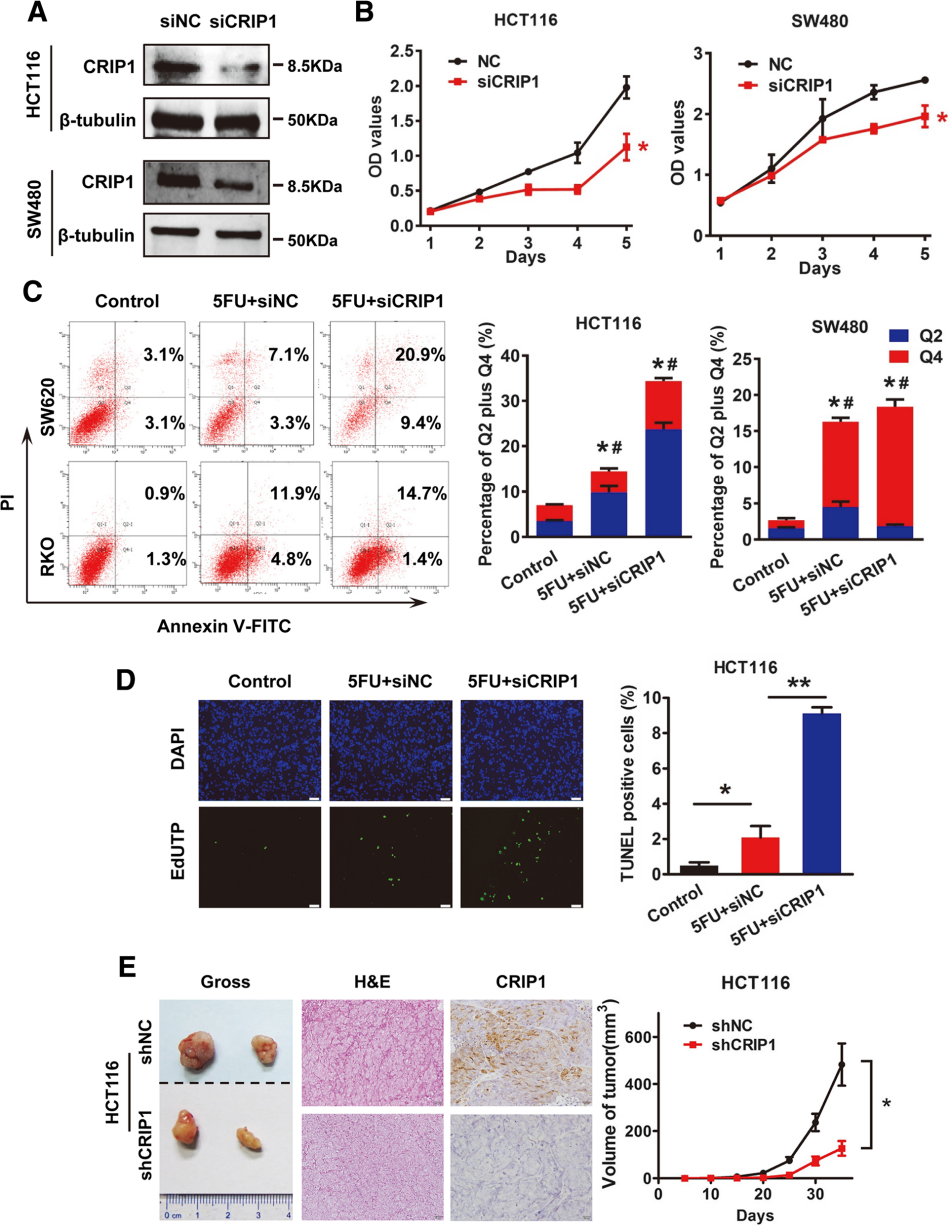
Knockdown of CRIP1 further suppresses 5-FU suppressed CRC cell proliferation both in vitro and in vivo*.* **a**. Western blot assay was used to detect the silencing of CRIP1 in HCT116 and SW480 cells. **b**. CCK8 assay was used to study the effects of siCRIP1 on 5-FU inhibited CRC cell proliferation. **c**. Annexin V-FITC/PI flow cytometry assay showed the effects of siCRIP1 on 5-FU induced apoptosis. Bars on the right panel represent percentage of cells in Q2 + Q4. **d**. TUNEL assay showed the effects of siCRIP1 on 5-FU induced apoptosis of CRC cells. Bars on the right panel represent the percentage of TUNEL positive cells. **e**. IHC staining showed the expression of CRIP1 in subcutaneous tumors formed by CRIP1 stable silencing CRC cells. Right panel represents the growth pattern of subcutaneous tumors

We silenced the expression of CRIP1 using siCRIP1 to confirm its effects on the proliferation and apoptosis of CRC cells. The silencing efficiency was tested by western blot assay (Fig. 3a). Cell proliferation was also evaluated by CCK8 assay after transfected with siCRIP1 in vitro. Compared with control, cell proliferation of HCT116 and SW480 cells in siCRIP1 group was dramatically reduced in CCK8 assay (*P* < 0.05; Fig. 3b). Consistently, siCRIP1 transient transfection did not affect the cell cycle of HCT116 cells (Additional file 1: Figure S1). Moreover, Annexin V-FITC/PI flow cytometry assay showed that silencing of CRIP1 further raised cell apoptosis rate of 5FU pre-treated HCT116 and SW480 Cells (*P* < 0.05; Fig. 3c). Apoptosis cells detected by TUNEL staining in green fluorescence also dramatically increased, which further confirmed the inhibitory role of CRIP1 in CRC cell apoptosis (*P* < 0.05; Fig. 3d).

CRIP1 stable silencing cells was constructed using shCRIP1 transfected HCT116 cells selected by puromycin to investigate the biological effect of CRIP1 in CRC cell proliferation in vivo. Silencing of CRIP1 significantly decreased the growth of subcutaneous tumors and the IHC signals of CRIP1 is weaker in the subcutaneous tumors formed by CRIP silencing CRC cells compared with that formed by the control group (Fig. 3e, Additional file 1: Figure S2, right panel), which further confirmed the stimulatory of CRIP1 on CRC proliferation in vivo.

### CRIP1 recovers 5-FU stimulated cell apoptosis by negatively regulating Fas-related pathway

**Fig. 4:**
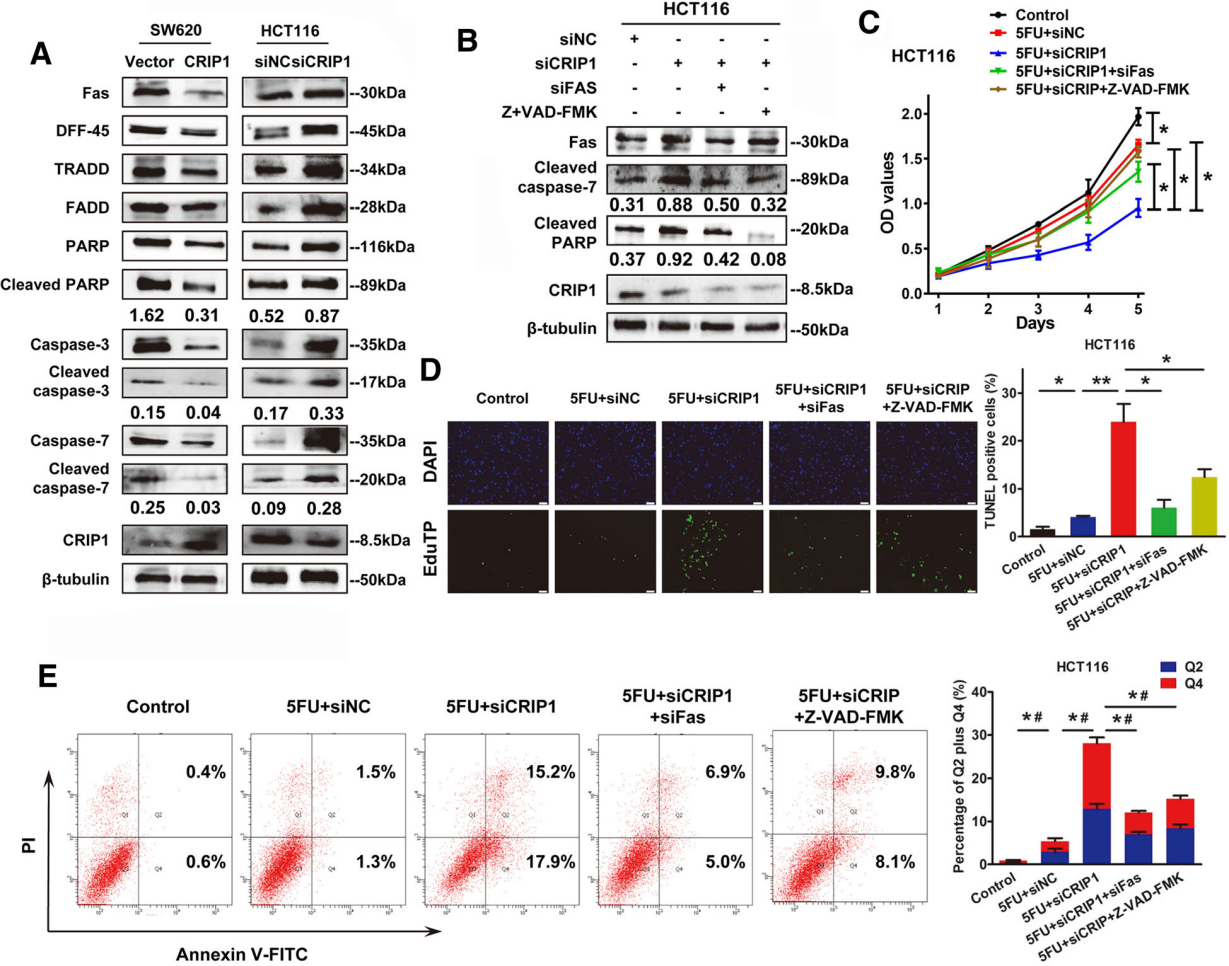
CRIP1 inhibits Fas induced apoptosis of CRC cells. **a**. Western blot analysis of apoptotic related proteins in CRIP1 overexpressed or silencing indicated cells. **b**. Western blot analysis of the effects of CRIP1 on Fas-mediated apoptosis. **c**. CCK8 assay shows the effects of CRIP1 on Fas-mediated apoptosis. **d**. Annexin V-FITC/PI flow cytometry assay showed the effects of CRIP1 on Fas-mediated apoptosis. Bars on the upper panel represent percentage of cells in Q2 + Q4. **e**. TUNEL assay showed the effects of CRIP1 on Fas-mediated apoptosis of CRC cells. Bars on the right panel represent the percentage of TUNEL positive cells

Since CRIP1 negatively regulated cell apoptosis, proteins related to apoptosis were detected by western blot assay in both CRIP1 overexpressing and silencing CRC cells. As revealed in Fig. 4a, proteins relevant to Fas mediated apoptotic signaling cascade including Fas, DFF-45, TRADD, FADD, cleaved PARP, cleaved caspase-3 and cleaved caspase-7 were down-regulated after transient overexpression of CRIP1. In contrast, silencing of CRIP1 up-regulated the expression of those proteins. Silencing of Fas could counteract the activation of PARP and caspase-7 induced by the silencing of CRIP1. The silencing efficiency of Fas was verified by western blot assay (Additional file 1: Figure S3). Z-VAD-FMK (50 μM), an irreversible inhibitor of caspase, could also restore the activation of PARP and caspase-7 stimulated by silencing of CRIP1 (Fig. 4b). CCK8 assay indicated that either knockdown of Fas or administration of Z-VAD-FMK dramatically recovered the suppressed cell proliferation caused by silencing of CRIP1 (Fig. 4c). Consistently, both flow cytometry assay and TUNEL assays showed that either knockdown of Fas or treatment with Z-VAD-FMK could remarkably suppress cell apoptosis induced by CRIP1 silencing (Fig. 4d, e, Additional file 1: Figure S4).

### CRIP1 interacts with Fas and enhances its ubiquitination and degradation

**Fig. 5:**
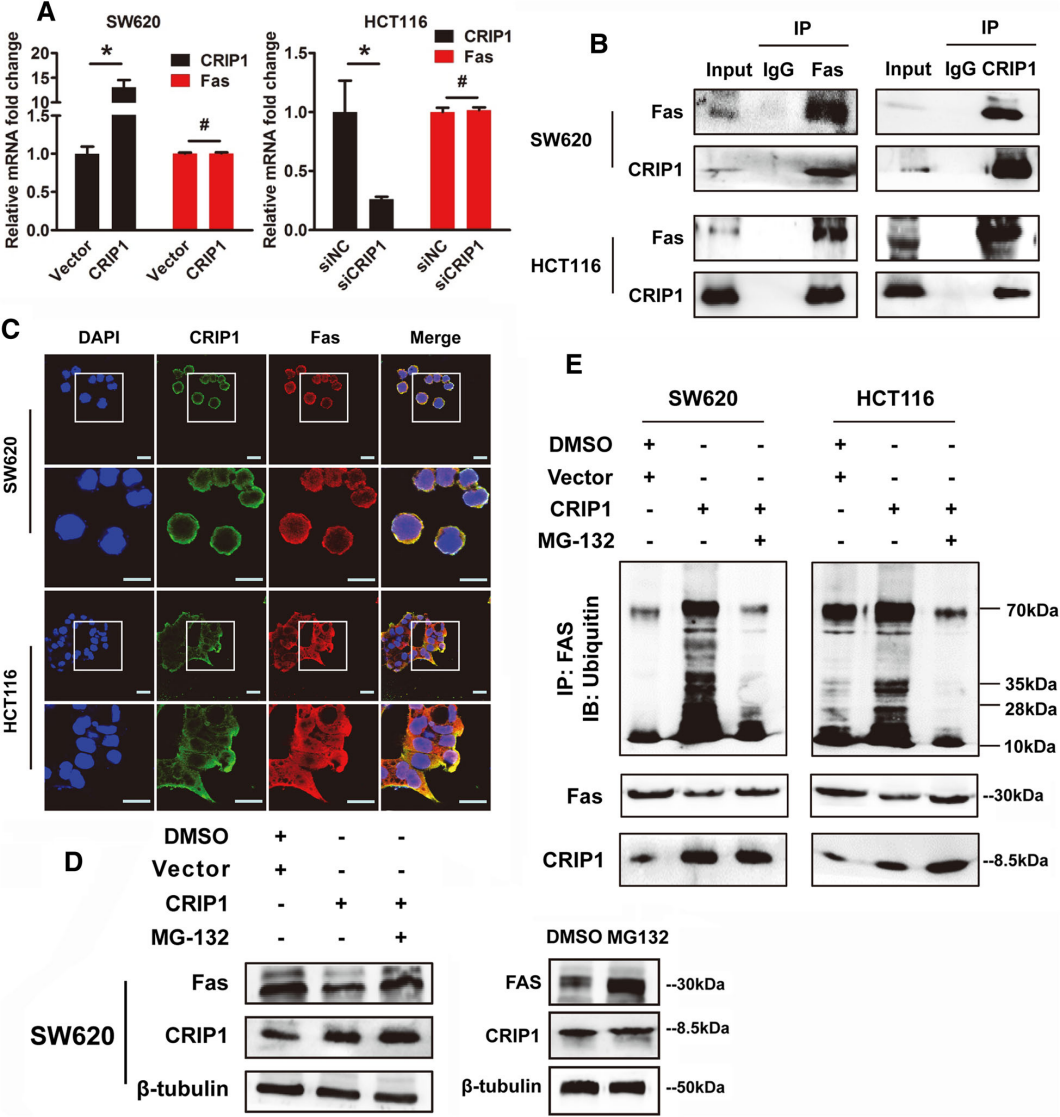
CRIP1 stimulates the ubiquitination and degradation of Fas. **a**. Real-time PCR shows the effects of CRIP1 on the expression of Fas at transcriptional level in both SW620 and HCT116 cells. **b**. Co-Ip shows the interaction between CRIP1 and Fas in both SW620 and HCT116 cells. **c**. The immunofluorescence staining demonstrated the co-localization of CRIP1 and Fas in both SW620 and HCT116 cells. **d**. Left panel: western blot assay shows that MG-132 recovered the inhibitory role of CRIP1 on Fas Protein. Right panel: the effects of MG-132 alone on the expression of Fas protein. **e**. CRIP1 stimulates the ubiquitination of Fas, while both siCRIP1 and MG-132 could recover the ubiquitin-mediated degradation of Fas

Real-time PCR were performed to evaluate the effect of CRIP1 on the expression of Fas at transcriptional level. However, no obvious difference was found in CRIP1 overexpression or silencing CRC cells compared with control cells (Fig. 5a). As obtaining the protein interaction domain, CRIP1 may function through protein-protein interaction. Protein pellets were purified from cell extraction by anti-CRIP1 or anti-Fas affinity gel and detected via western blot with anti-Fas or anti-CRIP1 antibodies. Interaction of the CRIP1 and Fas was further validated by co-Immunoprecipitation (Co-IP) assay using protein extraction of SW620 and HCT116 cells (Fig. 5b). Moreover, an obvious co-localization of CRIP1 and Fas was found by immunofluorescence in both CRC cells and CRC tissues (Fig. 5c, Additional file 1: Figure S5).

The Fas degradation induced by silencing of CRIP1 was totally recovered with the existence of MG132, which reduces the degradation of ubiquitin-conjugated proteins (Fig. 5d). When protein synthesis was inhibited by cycloheximide (CHX), introduction of CRIP1 could significantly shorten the half-life of Fas protein, which may be neutralized by MG132 treatment (Additional file 1: Figure S6). Moreover, the increased ubiquitination of Fas was detected in protein pellets purified by anti-Fas, and MG132 obviously diminished that in both SW620 and HCT116 cells (Fig. 5e). Nevertheless, CRIP1 could not influence the expression of FasL in translational levels detected by western blot assay and ELASIA assay, even pre-treated with 5-FU (Additional file 1: Figure S7). Our results supported that CRIP1 should play a vital role in reducing Fas mediated CRC apoptosis and stimulate the following CRC progression.

### CRIP1 and Fas is negatively correlated in both subcutaneous tumors and clinical CRC samples

**Fig. 6:**
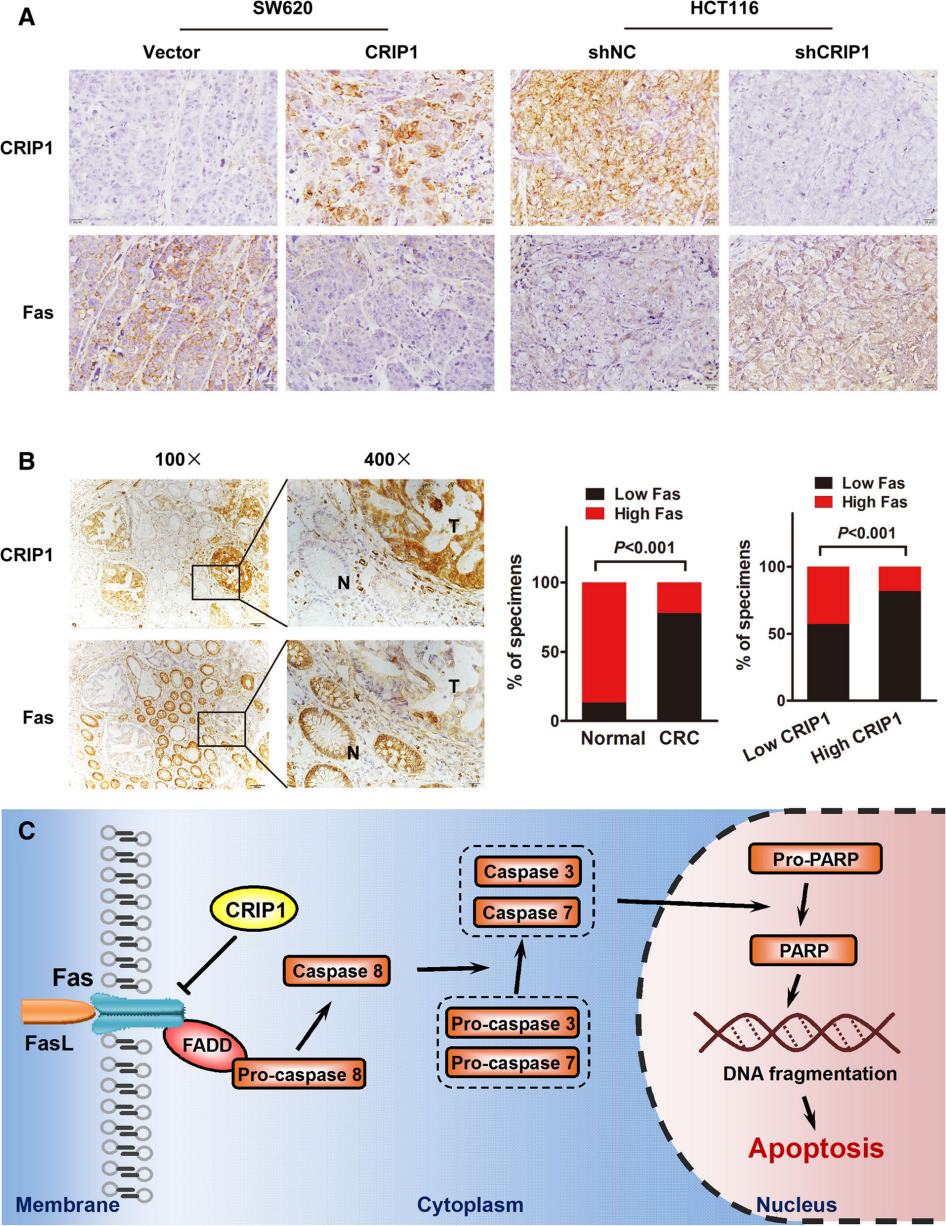
A negative correlation exists between the expression of CRIP1 and Fas in CRC tissues. **a**. Overexpression of CRIP1 is accompanied with the low expression of Fas, whereas silencing of CRIP1 is followed by the high expression of Fas in subcutaneous tumors. **b**. A reversed expression pattern of CRIP1 and Fas was detected in CRC tissues. Left panel: CRIP1 is usually overexpressed in CRC tissues while Fas highly expressed in its adjacent normal mucosa. The middle panel: the percentage of high or low Fas expression specimens in normal and cancer tissues. The right panel: the percentage of high or low Fas expression specimens in high or low CRIP1 expression specimens. **c**. A hypothetical model illustrating that CRIP1 interacts with Fas and stimulates the ubiquitin-mediated degradation of Fas to inhibit apoptosis of CRC cells

From the IHC staining of subcutaneous tumors formed by CRIP overexpression and silencing CRC cells, we found an obvious negative correlation between CRIP1 and Fas. Overexpression of CRIP1 in subcutaneous tumors is usually accompanied with the silencing of Fas, and vice versa (Fig. 6a). Studies on clinical samples of CRC patients further confirmed the similar relationship between CRIP1 and Fas. IHC assay revealed the negative correlation between the expression of CRIP1 and Fas in paraffin-embedded CRC sections. Using serial section, we found that Fas is highly expressed in adjacent non-tumor tissues while CRIP1 is overexpressed in CRC tissues under the same vision (R = 0.1905, *P* < 0.0001; Fig. 6b, left panel). Moreover, Fas was significantly down-regulated in 77.8% (35/45) of CRC tissues examined compared to the adjacent normal tissues (*P* < 0.001, Fig. 6b, middle panel). Fas was also significantly up-regulated in 42.9%(3/7) CRIP1 lowly expressed CRC tissues compared with 18.4% (7/38) CRIP1 highly expressed CRC tissues (Fig. 6b, right panel).

## Discussion

LIM family proteins have attracted extensive attentions for their presence of a cysteine riched LIM domain that has been demonstrated to direct protein-protein interactions involving in gene regulation, cell fate determination, tumour formation and cytoskeleton organization [22–25]. As a member of CRP subfamily of LIM family proteins, CRIP1 has been brought to the forefront due to its simple molecular structure and its clinical potential as a biomarker for cancer [26]. Although CRIP1 have been studied in various cancer types including prostate cancer, pancreatic caner, cervical cancer, breast cancer, osteosarcoma, thyoid carcinoma and gastric cancer. Unfortunately, functional studies of CRIP1 are limited and controversial. It seems that CRIP1 has tumor type specific oncogenic or tumor suppressive properties, and needs further clarification. Abbrent CRIP1 expression was also mentioned in colorectal cancer [27, 28], however its function in the development and progression of CRC remains largely unknown.

In our present study, we confirmed the ovexpression of CRIP1 in CRC tissues compared to the ajacent normal mucosa. Inconsisted with the newly published paper suggested that CRIP1 could not influence cell proliferation and apoptosis of CRC cells [28]. We found a stimulatory role of CRIP1 on CRC cell proliferation using two pairs of CRC cell lines. As the infinite proliferation of tumor cells attribute to the chemoresistance for tumor chemotherapy, the effects of CRIP1 on the proliferation and chemosensitivity of CRC cells were investigated. 5-FU, the cornerstone of CRC chemotherapy, is widely accepted as the first-line treatment for metastatic colorectal cancer (mCRC). Although these treatments provide regression in many cases, the relapse of cancer occurs due to the resistance of colorectal cancer cells to 5-FU. Therefore, 5-FU is used to uncover the effects of CRIP1 on anti-CRC drug resistance. Overexpression of CRIP1 dramatically recovered the inhibitory role of 5-FU on CRC cell proliferation and 5-FU induced apoptosis. In addition, CRIP1 counteracted the effects of 5-FU suppressed CRC cell proliferation through regulating CRC cell apoptosis. As CRIP1 is a small biomolecule that is easy to probe as a biomarker for cancer, mechanism exploration in-depth is meaningful for clinical therapy of CRC.

Apoptosis is a tightly controlled process that was recognized inducing via three major ways, including mitochondrial, endoplasmic reticulum and death receptor signaling pathway [29]. The extrinsic death receptor-mediated apoptotic signaling pathway was investigated and apoptotic cascade related proteins involving TRADD/FADD, Caspase-3, caspase-7, PARP and their cleaved forms were all down-regulated in CRIP1 overexpressed CRC cells. As a transmembrane protein that belongs to tumor necrosis factor superfamily, Fas is one of the most important uppermost stream proteins in most apoptosis signaling pathways. Fas ligand (FasL) can bind with the death domain of Fas to initiate apoptotic process. In the present study, we found that CRIP1 could dramatically reduce the expression of Fas protein without influencing the expression of its mRNA. Moreover, the co-localization of Fas and CRIP1 was also demonstrated in the membrane of CRC cells. Although the precise function is still illusive and controversial, CRIP1 was demonstrated to play an important role in orchestrating growth and differentiation probably through protein-protein interaction during transcriptional process, immune response and cytokine expression [30–32]. With the presence of the LIM domain that directing protein-protein interaction, CRIP1 was proposed directly interacted with Fas in CRC cells to enable the Fas mediated apoptotic cascade. Our hypothesis is confirmed by the Co-IP assay and the interaction between CRIP1 and Fas was verified. However, CRIP1 did not influence the expression of FasL in CRC cells. As FasL is predominantly expressed in activated T lymphocytes and Natural Killer (NK) cells that mediates cytotoxic apoptosis in the development of T/NK cells [33, 34], CRC derived CRIP1 might contribute to the regulation of FasL in the immune cells around the inflammatory environment of colorectal cancer.

As a key factor in cell apoptosis, Fas is closely associated with many biomolecules. MicroRNAs including miR-133b-5p, miR-25, miR-29c miR-196b and miR-221 were demonstrated inducing apoptosis by targeting Fas [35–38]. Met12 reduced apoptosis as a small peptide antagonist of Fas [39]. Thy-1 influenced the apoptosis of fibroblast by interacting with Fas protein in lipid rafts [40]. Our data revealed a novel biomolecules that could regulate the expression of Fas. CRIP1 could interact with Fas and reduced its expression at protein level. Ubiquitination is the most common way to degrade proteins in eukaryotes and widely takes part in various biological activities. Target protein is firstly labeled by ubiquitin, then recognized and degraded by proteasomes. Ubiquitin-mediated Fas degradation was proposed involving in CRIP1 regulated Fas expression. As expected, CRIP1 dramatically stimulated the ubiquitination of Fas, as well as the following protein degradation. The CRIP1 induced degradation of Fas was totally recovered with the existence of MG132, which reduces the degradation of ubiquitin-conjugated proteins. We treated cells with MG132 for only 6 h to avoid the interference of MG132 induced cell apoptosis. Although CRIP1 does not possess ubiquitin ligase activity, CRIP could influence the ubiquitination and degradation of Fas through interacting with ubiquitination related components via its LIM domain. An obvious negative correlation between CRIP1 and Fas was demonstrated from the IHC staining of subcutaneous tumors formed by CRIP overexpression and silencing CRC cells. Overexpression of CRIP1 in subcutaneous tumors is usually accompanied by the silencing of Fas, and vice versa. IHC of clinical samples further confirmed the relationship between Fas and CRIP1. We revealed that CRIP1 is predominantly expressed in the CRC tissues while Fas is mainly existence in the adjacent normal mucosa. High CRIP1 expression is usually accompanied by the low expression of Fas. CRIP1, undoubtedly, is a potential biomarker to predict the prognosis of CRC patients, especially with the involvement of Fas. Moreover, CRIP1 is a small molecule secretion protein that might be easily detected in the blood, which is probably considered as a noninvasive detection method for CRC in the future. As a multi-functional protein, CRIP1 might promote CRC cancer progression through other mechanisms. CRIP1 might also take part in the migration and metastasis of CRC for the high expression of CRIP1 in metastatic cell lines compared with that in non-metastatic cell lines. Further study is needed to reveal the roles of CRIP1 in CRC cells.

## Conclusions

In summary, this study revealed that CRIP1 is overexpressed in CRC tissues and suppresses chemosensitivity to 5-FU resistance via reducing Fas mediated apoptosis. As illustrated in the hypothetical model, CRIP1 interacts with Fas and stimulates the ubiquitin-mediated degradation of Fas to inhibit apoptosis of CRC cells (Fig. 6c). The reverse expression pattern of the two proteins was also verified in the IHC of clinical samples. Clinically, CRIP1 might be a promising therapeutic target and predictive factor for the chemotherapy of CRC.

## Additional file

Additional file 1: **Table S1.** The primers used in real-time PCR detection. **Figure S1.** Flow cytometry and EdU incorporation assay investigated the effects of CRIP1 on the cell cycle of CRC cells. **Figure S2.** Effects of CRIP1 on the growth of subcutaneous tumors. **Figure S3.** Western blot assay was used to detect the silencing efficiency of siRNA for FAS in HCT116 cells. **Figure S4.** Annexin V-FITC/PI flow cytometry assay showed the effects of Fas siRNA on 5-FU induced apoptosis in indicated cells. **Figure S5.** Immunofluorescence staining shows the localization of CRIP1 and Fas in CRC tissues. **Figure S6.** Fas expression was detected in CRIP1-overexpressing cells after MG132 or CHX treatment at different time. **Figure S7.** Effects of CRIP1 on the expression of Fas ligand (FasL). (DOCX 5746 kb)

## Abbreviations

5-FU: 5-Fluorouracil
CCK: Cell counting kit
CRC: Colorectal cancer
CRIP1: Cysteine-rich intestinal protein 1
FBS: Fetal bovine serum
IHC: Immunohistochemistry
NC: Negative control
PCR: Polymerase chain reaction
siRNA: Small-interfering RNAs
