# Supplementary material for: Cysteine-rich intestinal protein 1 suppresses apoptosis and chemosensitivity to 5-fluorouracil in colorectal cancer through ubiquitin-mediated Fas degradation
Source: J Exp Clin Cancer Res. 2019 Mar 8;38:120. doi: 10.1186/s13046-019-1117-z (PMC6408822; doi:10.1186/s13046-019-1117-z)
Supplement: Supplementary file 1 — Table S1. The primers used in real-time PCR detection. Figure S1. Flow cytometry and EdU incorporation assay investigated the effects of CRIP1 on the cell cycle of CRC cells. Figure S2. Effects of CRIP1 on the growth of subcutaneous tumors. Figure S3. Western blot assay was used to detect the silencing efficiency of siRNA for FAS in HCT116 cells. Figure S4. Annexin V-FITC/PI flow cytometry assay showed the effects of Fas siRNA on 5-FU induced apoptosis in indicated cells. Figure S5. Immunofluorescence staining shows the localization of CRIP1 and Fas in CRC tissues. Figure S6. Fas expression was detected in CRIP1-overexpressing cells after MG132 or CHX treatment at different time. Figure S7. Effects of CRIP1 on the expression of Fas ligand (FasL). (DOCX 5746 kb) [file 13046_2019_1117_MOESM1_ESM.docx]

**Additional file 1**

**Table S1. The primers used in real-time PCR detection.**

| Primer |  | Sequences |
| --- | --- | --- |
| GAPDH | Forward Primer | 5’-GGAGCGAGATCCCTCCAAAAT-3’ |
|  | Reverse Primer | 5’-GGCTGTTGTCATACTTCTCATGG-3’ |
| CRIP1 | Forward Primer | 5’-AATTCGGCACGAGGCATGATCCAA-3’ |
|  | Reverse Primer | 5’-AGAAGCCCCAGGAAAAGACTGACA-3’ |
| Fas | Forward Primer | 5’-GTGAGGGAAGCGGTTTACGA-3’ |
|  | Reverse Primer | 5’- AGATGCCCAGCATGGTTGTT-3’ |


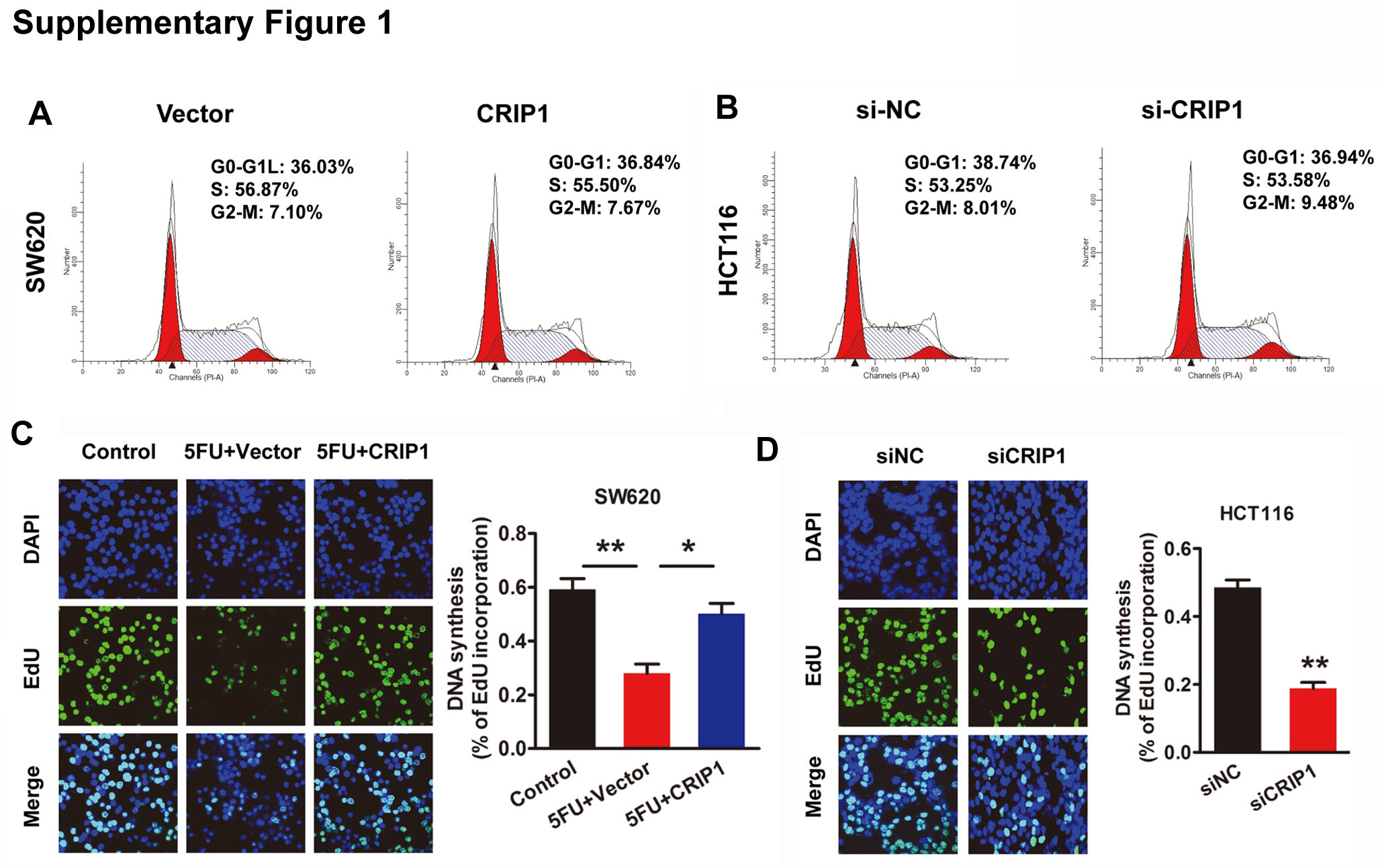


**Figure S1.** **Flow cytometry and EdU incorporation assay investigated the effects of CRIP1 on the cell cycle of CRC cells.** Upper panel: The effects of CRIP1 on the cell cycle of indicated cells were investigated by flow cytometry. Lower panel: EdU incorporation assay was used to study the effects of CRIP1 on 5-FU inhibited CRC cell proliferation. Bars on the left panel represent the quantified DNA synthesis.


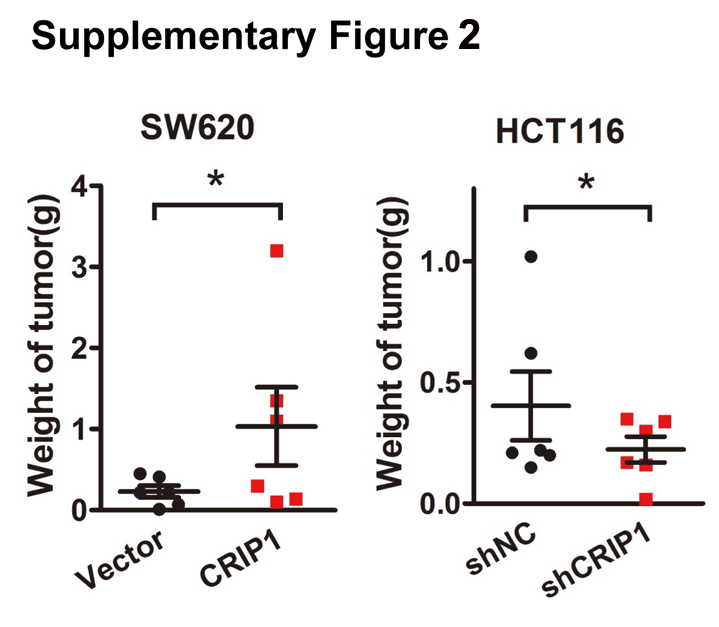


**Figure S2. Effects of CRIP1 on the growth of subcutaneous tumors.** Left panel: The weight of subcutaneous tumors formed by CRIP1 overexpressing CRC cells compared to the control CRC cells. Right panel: The weight of subcutaneous tumors formed by CRIP1 silencing CRC cells compared to the control CRC cells.


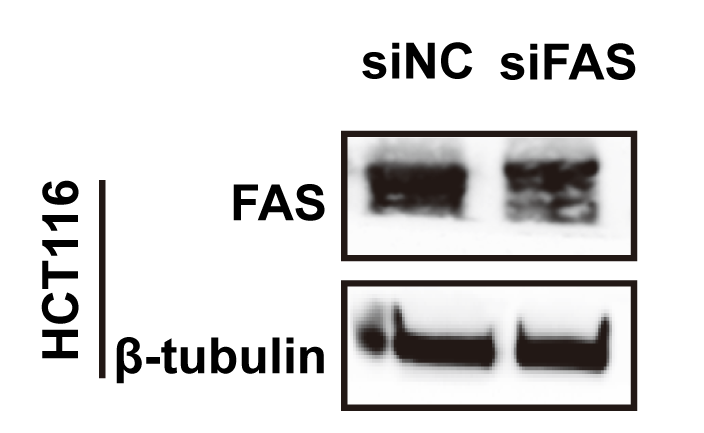


**Figure S3. Western blot assay was used to detect the silencing efficiency of siRNA for FAS in HCT116 cells.**


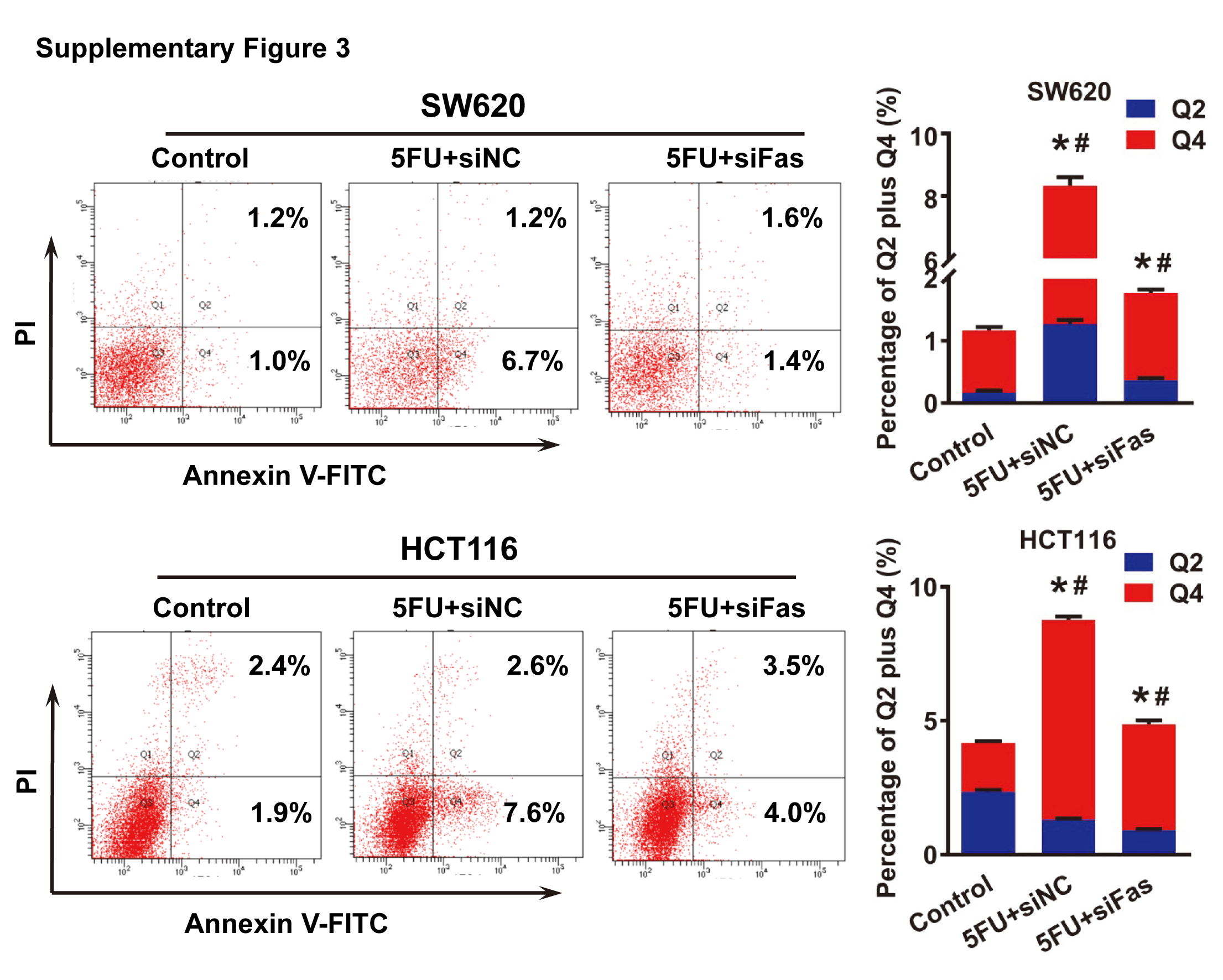


**Figure S4. Annexin V-FITC/PI flow cytometry assay showed the effects of Fas siRNA on 5-FU induced apoptosis in indicated cells.** Bars on the right panel represent percentage of cells in Q2+Q4.
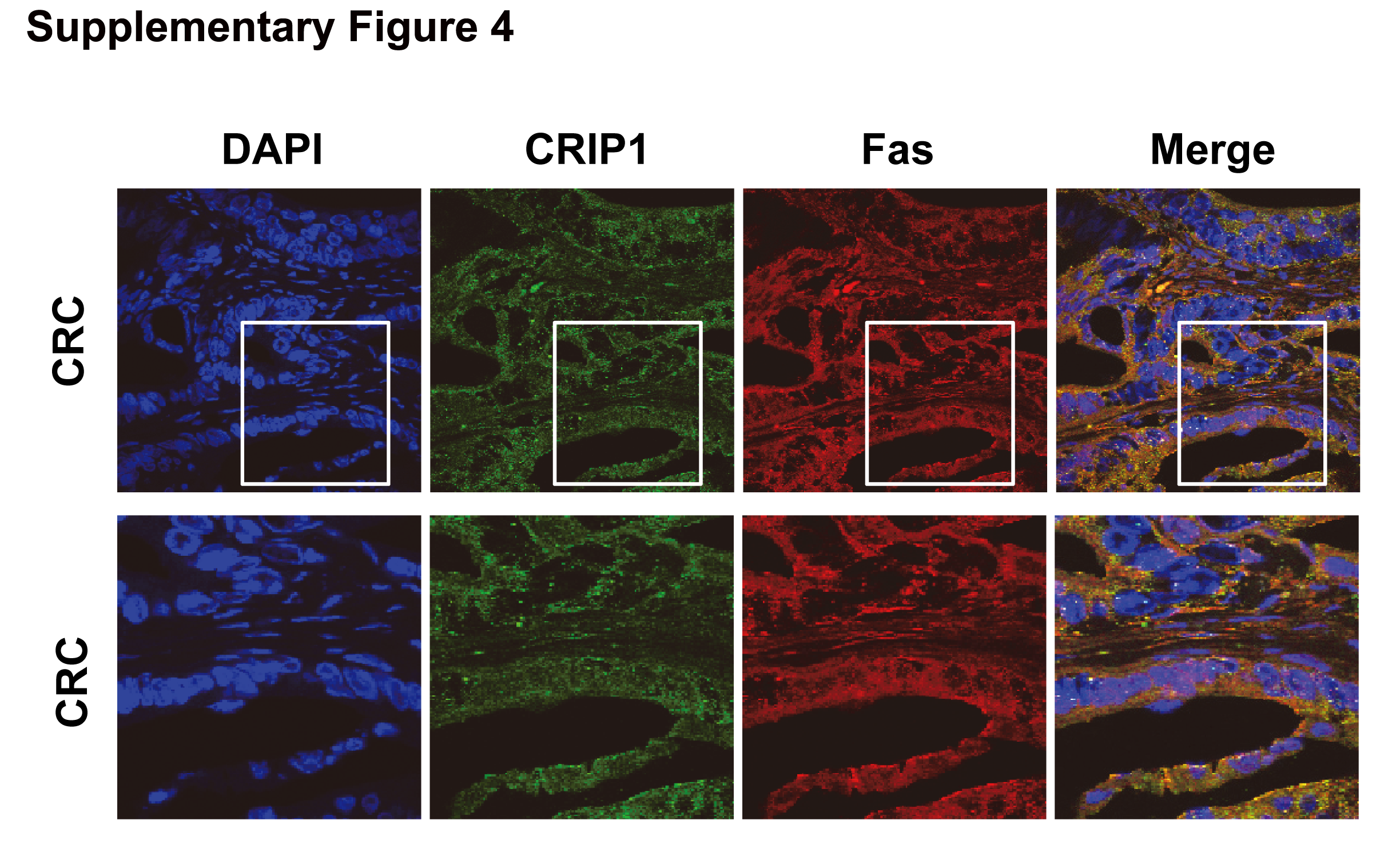


**Figure S5. Immunofluorescence staining shows the localization of CRIP1 and Fas in CRC tissues.** The lower panel is the amplification of the area selected in the upper panel.


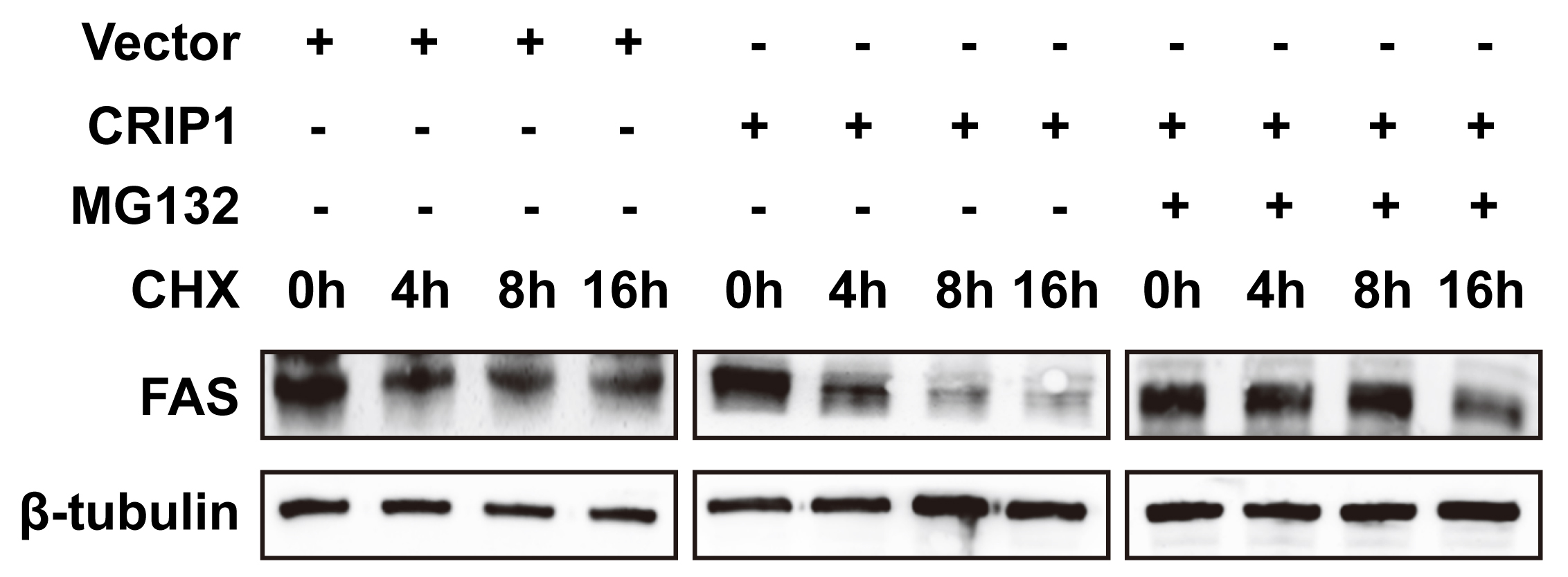
**Figure S6. Fas expression was detected in CRIP1-overexpressing cells after MG132 or CHX treatment at different time.**


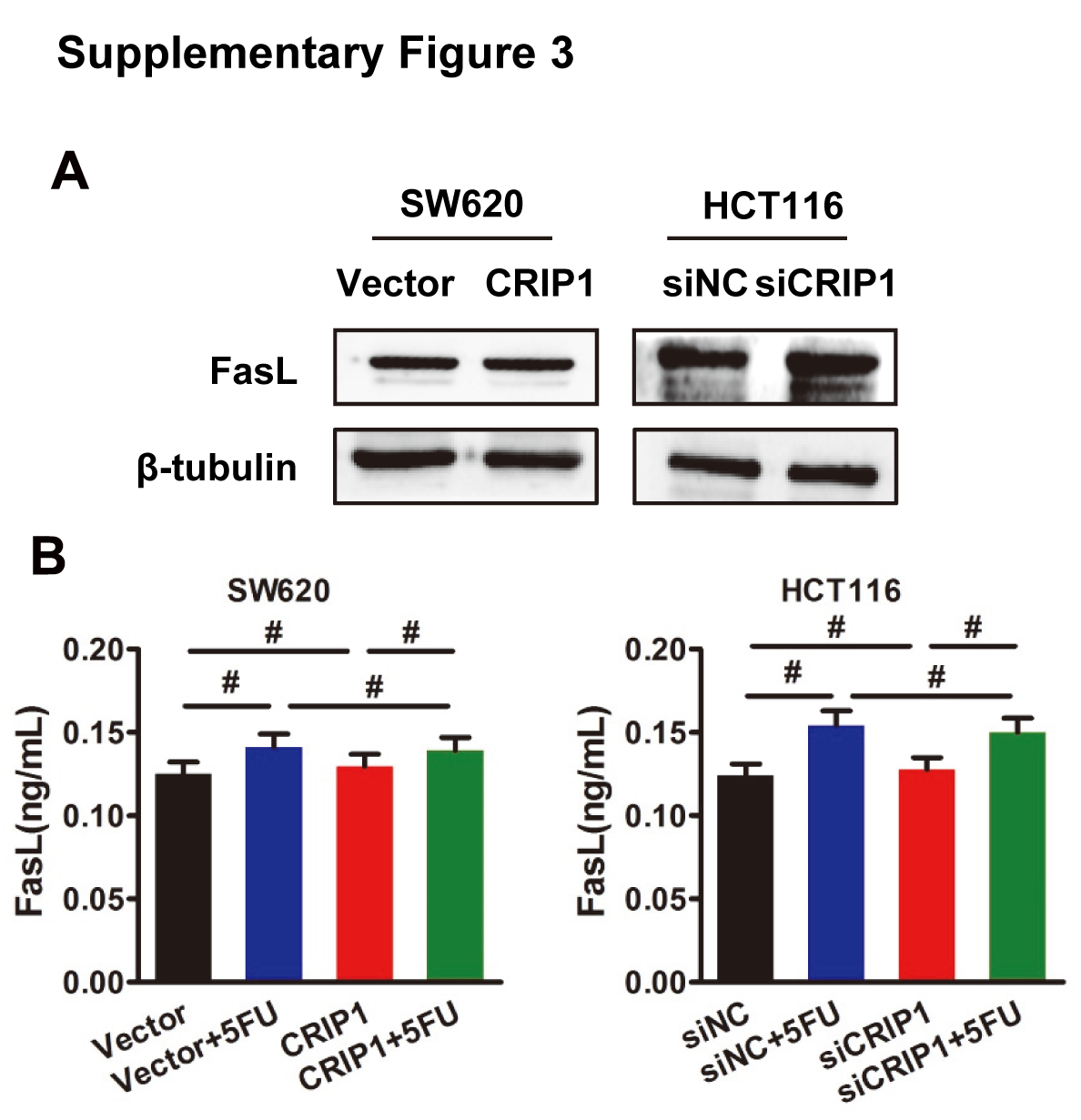


**Figure S7. Effects of CRIP1 on the expression of Fas ligand (FasL).** (A) Western blot analysis on the expression of FasL protein in CRIP1 overexpression or silencing CRC cells. (B) Enzyme linked immunosorbent assay (ELISA) analysis of FasL titer in CRIP1 overexpression or silencing CRC cells. ELISA detected the secretion of Fas in CRIP1 overexpressed SW620 and HCT 116 CRC cells w/o 5-FU pretreatment. Each bar represented the mean ± SD. The results were reproduced in three independent experiments. The asterisk (#) indicates P ＞ 0.05.
